# Compliance With New York City’s Beverage Regulations and Beverage Consumption Among Children in Early Child Care Centers

**DOI:** 10.5888/pcd11.130430

**Published:** 2014-10-16

**Authors:** Jakub Kakietek, Thearis A. Osuji, Sarah Abood O’Dell, Andrew Breck, Laura Kettel Khan

**Affiliations:** Author Affiliations: Thearis A. Osuji, Sarah Abood O’Dell, ICF International, Atlanta, Georgia; Andrew Breck, CDC Foundation, Atlanta, Georgia; Laura Kettel Khan, Centers for Disease Control and Prevention, Atlanta, Georgia.

## Abstract

**Introduction:**

This article examines the association between the New York City regulations on beverages served in child care centers and beverage consumption among enrolled children. The regulations include requirements related to beverages served to children throughout the day.

**Methods:**

Beverage consumption data were collected on 636 children enrolled in 106 group child care centers in New York City. Data on compliance with the regulations were collected through direct observation, interviews with center staff, and a site inventory. Logistic regression for rare events was used to test associations between compliance with the regulations and beverage consumption.

**Results:**

Compliance with the regulations was associated with lower odds of children consuming milk with more than 1% fat content and sugar-sweetened beverages during meals and snacks. There was not a significant relationship between compliance with the regulations and children’s consumption of water.

**Conclusion:**

The findings suggest a strong, direct relationship between what a center serves and what a child consumes, particularly regarding consumption of higher-fat milk and sugar-sweetened beverages. Therefore, policies governing the types of beverages served in child care centers may increase children’s consumption of more healthful beverages and reduce the consumption of less healthful ones.

## Introduction

Beverage consumption is a key factor in caloric intake that contributes to childhood obesity. Consumption of sugar-sweetened beverages (SSBs) such as sodas and fruit juice drinks with added sugar is associated with increased energy intake, overweight, and obesity in children (1–5). Although some research suggests that consumption of SSBs has decreased in the general population (5), most studies report that consumption of SSBs among children is increasing (6–8). Studies on milk consumption show varying effects on body mass index among children (9–13); however, the association between milk fat content and increased caloric intake has contributed to national guidelines supporting the consumption of low-fat and nonfat milk (14).

Child care centers have been identified as key settings to address childhood obesity (15,16). In response to evidence supporting the need for interventions in child care settings, in 2006 the New York City Department of Health and Mental Hygiene (DOHMH) modified regulations governing group child care centers. The modifications (amendments to Article 47 of the New York City Health Code) took effect in January 2007 and provided standards for what centers should offer in terms of beverages, physical activity, and screen time. The beverage-related regulations specify that 1) SSBs should not be provided to children, 2) milk served to children aged 2 or older should have a fat content of 1% or less, and 3) water must be readily available to children throughout the day.

This article contributes to the literature on policy interventions for obesity prevention by assessing the association between compliance with the New York City regulations and beverage consumption in children aged 3 or 4 years enrolled in a child care center in New York City.

## Methods

This evaluation involved a multimethod study design with 2 distinct data collection activities referred to as Center Evaluation Component and Class Evaluation Component (17). Center component data collection (October 2009–January 2010) focused on center-level measures. Class component data collection (April 2010–June 2010) focused on classroom- and child-level measures. Information on compliance collected in center and class components was intended to be complementary, and the study was not designed to compare compliance across methods.

### Study sample

The study population included the 1,654 child care centers licensed by the DOHMH Bureau of Child Care. Centers located in DOHMH District Public Health Offices (DPHO) catchment areas receive technical assistance and support services from the DOMH. Although most (301 of 311) of the DPHO catchment area child care centers were located in areas with high levels of poverty (census tracts with 40% or more of families with incomes at 200% of the federal poverty line or below), only 41% (549 of 1,343) of the non-DPHO centers were located in neighborhoods with high poverty levels. To ensure comparability between DPHO and non-DPHO centers, only centers in low-income, non-DPHO areas were included in the sampling frame. The final sampling frame included all 311 in DHPO neighborhoods and all 350 child care centers in 9 comparable low-income non-DPHO neighborhoods. Of the 260 centers randomly sampled, 26 (10%) were ineligible because they had an insufficient number of children (fewer than 10), had no children in the target age group (3- and 4-year-olds), enrolled only special-needs children, were closing or had already closed, or were unreachable. Of the 234 eligible centers, 58 (25%) refused to participate; 176 (75%) agreed to participate in the center component, and 110 (62.5%) agreed to participate in the class component. Complete beverage consumption data was collected on 636 children enrolled in 106 centers participating in the class component (17). Information on 24 children was missing for some meals in 4 centers. The centers were located in Manhattan, Bronx, Brooklyn, Queens, and Staten Island.

### Data collection

Center component data were collected during 2 site visits of 4 hours each. Data collection activities included computer-assisted, in-person interviews with child care center directors, teachers, and food service staff. Interviews documented center demographics, knowledge of the regulations, physical activity offered, food and beverages served, and training and technical assistance received. Data collectors also completed a structured site inventory to document child care center facilities and the beverages stored in kitchens or pantries. Data on training and technical assistance were provided by the New York City DOHMH.

Class component data were collected during 2-day site visits (approximately 8 hours per day during 2 consecutive days) in 1 randomly selected classroom per center of children aged primarily 3 or 4 years. Data collection included structured documentation of classroom activities, the types of foods and beverages served, staff characteristics and behavior, and child characteristics and behavior. Each day, 3 children in the classroom (for a total of 6 children per center) were randomly selected for observation of the amounts of foods and beverages served and consumed during meal and snack times. Data collectors reviewed the original beverage packaging (such as milk cartons, bottles, or juice boxes) and recorded all beverages served and consumed during the day. Data collection instruments were developed for this evaluation on the basis of existing validated instruments (18–21). Clearance was obtained from the ICF International institutional review board.

### Measures

**Compliance with New York City regulations.** Separate measures of compliance were constructed for each of the 3 components of the beverage regulations: milk, SSBs, and water. For each beverage type, compliance was measured by using a dichotomous indicator coded 1 if the center was compliant with the regulation on that beverage both at the center level (center component) and at the classroom level (class component) and 0 otherwise (compliant at the center level but not at the classroom level, compliant at the classroom level but not compliant at the center level, not compliant at either level).

In the center component, compliance with beverage regulations was determined by using data from the inventory of the child care center facilities. A center was coded compliant with the milk regulation if the center kitchen facilities contained only milk with 1% milk fat or less. A center was coded as compliant on the SSB regulation if beverages with added sweeteners (including artificial sweeteners) were not found in the center’s kitchen facilities. A center was considered compliant with the water regulation if teaching staff consistently reported that water was available to children throughout the day.

In the class component, compliance with beverage regulations was determined by classroom observation of the types of beverages served to children during meals and snacks. A center was considered compliant with regulation on milk if no milk with more 1% fat was served to any of the children with any meal or snack during the day. A center was considered compliant with the regulation on SSBs if no SSBs were served to any child with any meal or snack during the day. Finally, a center was considered compliant with the regulation on water if it had drinking water visible (eg, in pitchers or bottles) or a water fountain in the classroom or in a nearby hallway.

**Beverage consumption.** Consumption of milk with more than 1% fat, consumption of SSBs, and consumption of water were coded by using dichotomous indicators of whether a child consumed the beverage type (milk with more than 1% fat, SSB, water) at any meal during the day. Milk consumption was coded 1 if a child consumed milk with more than 1% fat with any meal or snack during the day in child care and 0 otherwise. SSB consumption was coded 1 if a child consumed an SSB with any meal or snack during the day in child care and 0 otherwise. Water consumption was coded 1 if a child consumed water with any meal or snack during the day in child care and 0 otherwise.

**Control variables.** Variables theorized to affect beverage consumption were considered as control variables; if these variables exhibited a significant bivariate relationship with the dependent variable of interest, they were included in the multivariate models. The following center-level variables were included in the analysis: center’s participation in 1) Child and Adult Care Food Program (CACFP) and 2) Head Start; 3) location inside or outside a DPHO catchment area; 4) average classroom size; 5) number of hours of operation per day; 6) student–teacher ratio; 7) annual teaching staff turnover rate; 8) participation in Eat Well Play Hard (EWPH); 9) the number of staff trained in EWPH Training of Trainers (TOTs); and 10) the number of TOTs-trained staff in the classroom of observation. The number of meals the observed child consumed during the day of observation was included to account for increased likelihood of consuming any beverage, including those prohibited by the regulations, by children who had more meals during the day compared with those who had fewer meals (eg, refused to have some of the meals provided by the center).

### Analysis

Multiple regression analysis was used to assess the association between compliance and beverage consumption while controlling for potential confounding factors. Because most children did not consume prohibited beverages, the distribution of the dependent variables was skewed toward 0. To correct for potential bias and inefficiency of the logistic regression resulting from a zero-inflated sample, logistic regression for rare events was used (22). All analyses were conducted using Stata 11 (StataCorp LP).

As a robustness check, we re-estimated all models using logistic regression with robust standard errors, which corrects for inefficiency of the estimates due to clustering (23,24). The results obtained by using logistic regression with robust standard errors were virtually the same as the results obtained by using rare event logistic regression.

## Results

An average center in the sample had about 16 children per classroom and 6 children per teacher and was open for about 10 hours per day. An average center participated in one training related to nutrition other than EWPH. In an average center and in an average classroom, fewer than 1 teacher participated in TOTs (Table 1). Most (59.3%) centers were located in the DPHO catchment area; 87.7% participated in CACFP, 68.9% participated in Head Start, and 39.6% participated in EWPH (Table 1).

**Table 1:** Characteristics of 106 Child Care Centers, New York City, 2010

Variable	Value
**Continuous, mean (SD) **	
Average classroom size, no. of children, range, 7–27	16.5 (3.5)
Operating hours, hours/day, range, 6.75–13.5	10.0 (1.0)
Student–teacher ratio, range, 0.30–22	5.8 (3.0)
Teaching staff turnover, range, 0–0.80	0.08 (0.13)
No. of nutrition-related training programs other than Eat Well Play Hard in which the center participated, range, 0–2^a^	0.77 (0.62)
No. of staff in the center who participated in Training of Teachers, range, 0–23^b^	0.55 (2.41)
No. of staff in the classroom who participated in Training of Teachers, range, 0–3^b^	0.66 (0.93)
No. of meals the child had during the day of observation, range, 2–4	3.05 (0.35)
**Categorical, no. (%)**	
Center was in a District Public Health Office^c^ catchment area	63 (59.3)
Center participated in Child and Adult Care Food Program^d^	93 (87.7)
Center participated in Head Start^e^	73 (68.9)
Center participated in Eat Well Play Hard^a^	42 (39.6)

a Eat Well Play Hard is a technical assistance program of the New York City Department of Health and Mental Hygiene that teaches staff and children about nutrition and physical activity.

b Eat Well Play Hard Training of Teachers is a technical assistance program of the New York City Department of Health and Mental Hygiene that teaches child care center staff how to lead the Eat Well Play Hard nutrition and physical activity curriculum in their classrooms.

c District Public Health Offices are a program of the New York City Department of Health and Mental Hygiene that target resources to high-need neighborhoods in the South Bronx, East and Central Harlem, and North and Central Brooklyn. These centers all received 2 sessions of individualized on-site technical assistance. Only 97 centers were counted.

d The Child and Adult Care Food Program is a program of the United States Department of Agriculture that administers federal grants to state health departments to provide nutritious meals and snacks to low-income individuals.

e Head Start is a comprehensive developmental program for preschool aged children and their families who earn household income below the federal income poverty threshold administered by the Administration for Children and Families within the United States Department of Health and Human Services.

Overall, the level of compliance with the regulations in the centers was high (Table 2). Most centers (75.5%) complied with the milk regulation, and 67% of the centers did not serve SSBs. Water was available to children in 52.8% of the centers. All centers in the sample provided meals to children. The number of centers where children consumed beverages brought from home varied by meal type and day of observation. Most centers (92 centers, 83.6%) did not allow any food from home. Only 2 centers (1.9%) allowed foods from home for all eating occasions. The other 16 allowed it at some eating occasions.

**Table 2:** Compliance^a^ With the New York City Regulation on Milk, SSBs, and Water and Beverage Consumption in 636 Children in 106 Child Care Centers, New York City, 2010

Measure	No. (%)
**Compliance with New York City regulations (n = 106)**	
**Milk served to children 2 years and older should have a fat content of 1% or less**	
Compliant	80 (76.4)
Not compliant	26 (24.5)
**SSBs should not be provided to children (n = 106)**	
Compliant	70 (66.7)
Not compliant	36 (33.3)
**Water must be readily available to children throughout the day (n = 106)**	
Compliant	56 (51.8)
Not compliant	50 (41.2)
**Beverage consumption (n = 636)**	
**Child consumed milk with more than 1% fat**	
No	576 (90.6)
Yes	60 (9.4)
**Child consumed SSBs**	
No	552 (86.8)
Yes	84 (13.2)
**Child consumed water**	
No	464 (72.9)
Yes	172 (27.0)

Abbreviation: SSBs, sugar-sweetened beverages.

a See Methods section for a description of measures of compliance.

Most children in the sample did not consume beverages prohibited by the regulations: 90% did not consume milk with more than 1% fat, and 86.8% did not consume SSBs (Table 2). Only 27% of the children consumed water with meals or snacks.

Logistic regression analyses showed that compliance with regulations was associated with lower likelihood that a child consumed milk with more than 1% fat content or SSBs. Compliance was not associated with the likelihood of the child consuming water during meals or snacks (Table 3).

**Table 3:** Logistic Regression for Rare Events Models of the Association Between Compliance With New York City Regulations and Beverage Consumption^a^, Children in 106 Child Care Centers, New York City, 2010

Variable	OR (95% CI)		
	Model 1: Milk (N = 636)	Model 2: SSBs (N = 636)	Model 3: Water (N = 636)
Compliance^a^	0.03 (0.01–0.09)	0.14 (0.07–0.26)	0.70 (0.46–1.08)
Child and Adult Care Food Program^b^	0.42 (0.18–0.94)	0.41 (0.20–0.83)	0.22 (0.12–0.40)
Head Start^c^	Dropped	0.18 (0.07–0.43)	2.75 (1.74–4.34)
Center in a District Public Health Office catchment area^d^	1.43 (0.62–3.31)	0.90 (0.42–1.93)	1.68 (1.01–2.79)
Average classroom size^e^	1.04 (0.94–1.14)	0.81 (0.74–0.87)	0.93 (0.87–1.00)
Operating hours^e^	4.27 (2.64–6.89)	1.39 (0.96–1.99)	1.30 (1.03–1.65)
Student–teacher ratio^e^	0.92 (0.81–1.06)	0.95 (0.84–1.07)	0.95 (0.89–1.03)
Teaching staff turnover^e^	0.16 (0.01–1.66)	0.16 (0.02–1.12)	4.89 (1.11–21.47)
Center participated in Eat Well Play Hard^f^	4.54 (1.89–10.9)	2.44 (1.08–5.49)	1.33 (0.80–2.22)
No. of nutrition-related training programs other than Eat Well Play Hard^f^ in which the center participated	0.42 (0.24–0.72)	0.28 (0.14–0.55)	1.26 (0.92–1.72)
No. of staff in the center who participated in Training of Teachers^g^	1.00 (0.77–1.30)	0.67 (0.44–1.02)	1.04 (0.96–1.12)
No. of staff in the classroom who participated in Training of Teachers^g^	0.38 (0.14–1.05)	0.99 (0.62–1.56)	0.95 (0.73–1.24)
No. of meals the child had during the day of observation	5.97 (2.17–16.38)	2.85 (1.36–5.95)	3.62 (2.13–6.14)

Abbreviations: SSBs, sugar-sweetened beverages; OR, odds ratio; 95% CI, 95% confidence interval.

a Noncompliant at center level, classroom level, or both levels is the reference category. See Methods for a description of measures of compliance.

b The Child and Adult Care Food Program is a program of the United States Department of Agriculture that administers federal grants to state health departments to provide nutritious meals and snacks to low-income individuals.

c Head Start is a comprehensive developmental program for preschool-aged children and their families who earn household income below the federal income poverty threshold administered by the Administration for Children and Families in the Department of Health and Human Services (HHS). This variable was dropped from the model because none of the Head Start centers were noncompliant.

d District Public Health Offices are a program of the New York City Department of Health and Mental Hygiene that target resources to high-need neighborhoods in the South Bronx, East and Central Harlem, and North and Central Brooklyn. These centers all received 2 sessions of individualized on-site technical assistance.

e All continuous variables in the model are mean-centered.

f Eat Well Play Hard is a technical assistance program of the New York City Department of Health and Mental Hygiene that teaches staff and children about nutrition and physical activity.

g Eat Well Play Hard Training of Teachers is a technical assistance program of the New York City Department of Health and Mental Hygiene that teaches child care center staff how to lead Eat Well Play Hard nutrition and physical activity curriculum in their classrooms.

**Milk (Model 1).** Children in centers that were compliant with the milk regulation had 97% lower odds of consuming milk with more than 1% fat with any meal or snack than children in centers that were not compliant (adjusted odds ratio [AOR], 0.03; 95% CI, 0.01–0.09). Children in centers that participated in CACFP and in a greater number of training programs (beyond EWPH) were less likely to consume milk with more than 1% fat than children in centers that did not participate in the program (Table 3). Longer operating hours, center’s participation in EWPH, and greater number of meals and snacks were associated with greater likelihood that the child consumed milk with more than 1% fat. The indicator of center’s participation in Head Start was dropped from the regression model because none of the children in centers that participated in Head Start consumed milk with more than 1% fat.

**Sugar-sweetened beverages (Model 2).** Children in centers compliant with the SSB regulation had 86% lower odds of consuming SSBs with any meal or snack than children in centers that were not compliant (AOR, 0.14; 95% CI, 0.07–0.26). Center’s participation in CACFP and Head Start, the higher number of nutrition-related training programs other than EWPH, and larger classroom size were associated with lower likelihood that the child consumed SSBs (Table 3). A greater number of meals or snacks the child had during the day and center’s participation in EWPH were associated with a higher likelihood that SSBs were consumed with a meal or a snack.

**Water (Model 3):** Compliance with the water regulation was not associated with the likelihood of the child consuming water with a meal or a snack during the day (AOR, 0.70; 95% CI, 0.46–1.08). Factors associated with higher likelihood that the child consumed water with a meal or a snack were center’s participation in Head Start, location in a DHPO area, longer operating hours, greater teaching staff turnover, and greater number of meals and snacks during the day (Table 3). In contrast, center’s participation in CACFP was associated with lower likelihood that the child consumed water with a meal or a snack.

## Discussion

The literature on beverage consumption in child care settings focuses either on the assessments of nutritional content and quality of foods and beverages consumed in child care, often comparing them with national guidelines and standards (25–28) or on reviews comparing regulations in different jurisdictions (29,30). This evaluation bridges the gap between those 2 strands of research and directly examines the link between regulations and consumption. Studies have reported the effectiveness of regulatory approaches in school-aged children (31,32). Ours is the first study to examine the association of these types of interventions on children’s beverage consumption in child care centers in the United States.

Our findings show a strong association between center compliance with the New York City regulations and the types of beverages children consumed. Children in centers compliant with the regulations were less likely to consume SSBs and milk with more than 1% fat content than children in noncompliant centers. The results are encouraging and suggest that regulations prohibiting unhealthful beverages have the potential to limit consumption of these beverages among children in child care settings.

Our evaluation found that relatively few centers served unhealthful beverages (23.6% served milk with more than 1% fat, and 33.0% served SSBs). Because we wanted to create as restrictive a measure of compliance as possible, our measure may underreport compliance with the milk regulation. Specifically, we considered a center noncompliant with the regulation when milk with more 1% fat was found in the kitchen facilities. In centers serving children younger than 2 years, milk with more than 1% fat may have been stored on their premises but it was not served to children older than 2.

The degree to which less healthful beverages are served in child care settings varies. A recent study of child care centers in North Carolina found that 8% of the centers in the study sample served SSBs but as many as 50% served whole milk to children aged 3 to 5 (26). Studies conducted in New York City (29) and Georgia (33) found that water was available to children in about 50% and 55% of the centers, respectively, which is consistent with our findings (48%).

We did not find a significant association between compliance with the water regulation and the likelihood that the child consumed water with meals or snacks. Data collection on consumption of beverages was conducted only during meals and snacks, whereas the New York City regulations aimed at increasing the consumption of water throughout the day including meal and snack times. Because of the intensive and intrusive nature of data collection during 8 hours in a classroom, we focused on observation of consumption at meal times.

Despite that limitation, our findings are similar to those reported in the literature. A study of Connecticut child care centers found that despite policies promoting the availability and accessibility of water in child care centers and the availability of water in most classrooms, children were not prompted to drink water or the water was accessible only to adults (34). These data suggest that education and support on water consumption may be needed to facilitate greater compliance with regulations.

Consistent with the literature, participation in CACFP and Head Start was associated with improved beverage-related outcomes (35,36). Participation in Head Start was associated with lower odds that children consumed SSBs and greater odds that a child consumed water with any meal or snack. Furthermore, in centers participating in Head Start, none of the children consumed milk with more than 1% fat. Participation in CACFP was associated with lower odds that children consumed SSBs, but, unlike participation in Head Start, it was also associated with lower odds that the child consumed water with any meal or snack. This finding is consistent with the requirements of the 2 programs: Head Start guidelines encourage water consumption and, before 2011, CACFP guidelines encouraged the provision of low-fat milk at all mealtimes and recommended that water not be placed on the table during meal times for children aged 3 to 5 years (the CACFP policy was revised in 2011 and allows for water on the table during meals) (37,38).

One limitation of our study is the use of a cross-sectional design that does not establish cause between the adoption of the regulations and child-level outcomes. However, we did find an association between compliance with the types and frequency of beverages that children consumed. We are not aware of any systematic assessment of the consumption of SSBs, milk, or water in child care centers in New York City before the adoption of the regulation and cannot assess, even indirectly, any differences in day care center consumption after the introduction of the regulations. Another limitation of the study is that the data related to compliance with the New York City regulations at the center level is, in part, based on staff self-reports. To minimize the measurement bias, when possible, we used site inventories to capture center-level compliance. Furthermore, we captured classroom-level compliance through direct observations. Combining data from both sources strengthens the robustness of our compliance measure.

This study considered only beverages consumed during the child care center day and excluded beverages consumed in the home. As much as 70% of sugar-sweetened beverages may be consumed in the home (38). Nevertheless, within the limitations of the study setting, our findings highlight the potential effectiveness of policy interventions in child care centers and illustrate the need for further exploration of the relationship between regulations and consumption of less healthful beverages. 
